# Macromolecular crowding and decellularization method increase the growth factor binding potential of cell-secreted extracellular matrices

**DOI:** 10.3389/fbioe.2023.1091157

**Published:** 2023-01-23

**Authors:** Shierly W. Fok, Robert C. H. Gresham, Weston Ryan, Benjamin Osipov, Chelsea Bahney, J. Kent Leach

**Affiliations:** ^1^ Department of Orthopaedic Surgery, UC Davis Health, Sacramento, CA, United States; ^2^ Steadman Philippon Research Institute, Vail, CO, United States; ^3^ Department of Biomedical Engineering, University of California, Davis, Davis, CA, United States

**Keywords:** macromolecular crowding, decellularization, BMP-2, bone, growth factors

## Abstract

Recombinant growth factors are used in tissue engineering to stimulate cell proliferation, migration, and differentiation. Conventional methods of growth factor delivery for therapeutic applications employ large amounts of these bioactive cues. Effective, localized growth factor release is essential to reduce the required dose and potential deleterious effects. The endogenous extracellular matrix (ECM) sequesters native growth factors through its negatively charged sulfated glycosaminoglycans. Mesenchymal stromal cells secrete an instructive extracellular matrix that can be tuned by varying culture and decellularization methods. In this study, mesenchymal stromal cell-secreted extracellular matrix was modified using λ-carrageenan as a macromolecular crowding (MMC) agent and decellularized with DNase as an alternative to previous decellularized extracellular matrices (dECM) to improve growth factor retention. Macromolecular crowding decellularized extracellular matrix contained 7.7-fold more sulfated glycosaminoglycans and 11.7-fold more total protein than decellularized extracellular matrix, with no significant difference in residual DNA. Endogenous BMP-2 was retained in macromolecular crowding decellularized extracellular matrix, whereas BMP-2 was not detected in other extracellular matrices. When implanted in a murine muscle pouch, we observed increased mineralized tissue formation with BMP-2-adsorbed macromolecular crowding decellularized extracellular matrix *in vivo* compared to conventional decellularized extracellular matrix. This study demonstrates the importance of decellularization method to retain endogenous sulfated glycosaminoglycans in decellularized extracellular matrix and highlights the utility of macromolecular crowding to upregulate sulfated glycosaminoglycan content. This platform has the potential to aid in the delivery of lower doses of BMP-2 or other heparin-binding growth factors in a tunable manner.

## 1 Introduction

Growth factors are potent bioactive macromolecules used for tissue repair due to their capacity to induce mitogenesis, chemotaxis, differentiation, and instruct cell fate (Gresham et al., 2021). Numerous growth factors have been investigated for tissue regeneration including bone morphogenetic protein-2 (BMP-2) for spinal fusion. The INFUSE^®^ bone graft (Medtronic, Inc.) is the most well established osteoanabolic approach, delivering milligram quantities of BMP-2 from an absorbable collagen sponge (McKay et al., 2007). However, INFUSE suffers from widespread reports of off-target and adverse systemic effects due to unregulated BMP-2 diffusion and the large quantities of costly growth factors required (Carragee et al., 2011; James et al., 2016). Thus, there is a critical need to develop improved delivery methods to realize the therapeutic potential of BMP-2 and other growth factors for tissue regeneration.

While a host of natural and synthetic platforms have been investigated to control the presentation of growth factors including BMP-2 (Ren et al., 2019; Gresham et al., 2021), the native extracellular matrix (ECM) functions as a reservoir for endogenous growth factors and provides adhesion sites for associated cells. Mesenchymal stromal cells (MSCs) secrete a complex ECM enriched in matricellular proteins and glycosaminoglycans that can function as an instructive biomaterial for osteogenic differentiation (Harvestine et al., 2018), and we demonstrated that the quantity and composition of this ECM can be controlled by the cell culture conditions (Decaris and Leach, 2011; Decaris et al., 2012). This cell-secreted ECM consistently increases cell survival (Hoch et al., 2016), induces or retains the osteogenic phenotype of MSCs (Bhat et al., 2011; Harvestine et al., 2018; Harvestine et al., 2020), and enhances the responsiveness of associated MSCs to soluble inductive cues (Gonzalez-Fernandez et al., 2022). Due to the innate capacity of the ECM to retain growth factors and present them in a biological context, cell-secreted ECM is a promising platform for growth factor presentation for tissue regeneration. Therapeutic application of ECM requires decellularization to avoid immunogenicity, yet decellularization can impact the protein composition and structure (Gilbert et al., 2006; White et al., 2017). Specifically, common decellularization methods eliminate essential heparan sulfate proteoglycans from the ECM (Crapo et al., 2011), which are essential for adsorption of heparin-binding growth factors.

Physiologically, cells reside in crowded microenvironments. Previous studies incorporated macromolecules as an *in vitro* biomimetic strategy to recapitulate native conditions and assess crowding effects on protein synthesis (Prewitz et al., 2015; Marinkovic et al., 2021). Macromolecular crowding (MMC) employs macromolecules to occupy volume, increasing the effective concentration of other macromolecules that promote supramolecular assembly, boosting enzyme activity through the stabilization of enzyme-substrate complexes, and reducing the lag phase of protein synthesis (Dewavrin et al., 2015; Rivas and Minton, 2016; Badowski et al., 2020). MMC induces collagen fiber formation through the upregulation of fibronectin fiber assembly, resulting in increased collagen nucleation sites (Graham et al., 2019). MMC agents such as Pluronic (Guerzoni et al., 2022), Ficoll™ (Lareu et al., 2007), dextran sulfate (Assunção et al., 2020), fucoidan (Yoo et al., 2022), and polydisperse carrageenan (Satyam et al., 2016) have been studied for their influence on the ECM for applications in tendon and tissue equivalents (Cigognini et al., 2016; Tsiapalis et al., 2020; Marinkovic et al., 2021). Herein, this study focuses on λ-carrageenan, a red seaweed-derived polysaccharide often used as an emulsifier. Carrageenan supplementation induces notable increases in sGAG, making it a promising MMC for tuning the composition of a cell-secreted ECM. The use of λ-carrageenan avoids media viscosity increases reported with other carrageenan isoforms (Cigognini et al., 2016). While prior studies assessed the influence of MMC agents on protein synthesis, the influence of λ-carrageenan MMC and decellularization technique on the function of decellularized ECM for bone formation has not been reported.

We hypothesized that λ-carrageenan, when used as a MMC agent, would enhance the production of sulfated glycosaminoglycan (sGAG) in MSC-secreted ECM. We further hypothesized that sGAGs could be retained in cell-secreted ECM by modifying the decellularization protocol, resulting in a platform that offers improved endogenous growth factor retention and exogenous growth factor adsorption and presentation. We evaluated the effects of λ-carrageenan and decellularization methods on MSC-secreted ECM composition, sGAG content, and endogenous BMP-2 retention. We then tested the ability of MMC dECM to adsorb and release BMP-2 to induce mineralized tissue formation.

## 2 Materials and methods

### 2.1 Cell culture

Human bone marrow-derived mesenchymal stromal cells (MSCs, Lot# 00238, RoosterBio, Frederick, MD) were expanded until passage 5 in standard growth media composed of minimum essential medium alpha ( α MEM, Life Technologies, Carlsbad, CA) with 10% fetal bovine serum (FBS, Genesee Scientific, San Diego, CA) and 1% Penicillin-Streptomycin (P/S, Gemini, Sacramento, CA). MSCs were maintained in standard culture conditions (21% O_2_, 5% CO_2_, 37 ° C) and used at passage 5-6. The BMP Responsive Immortalized Reporter (BRITER) osteoblast cell line (Kerafast, Boston, MA) isolated from murine calvaria was cultured in Dulbecco’s Modified Eagle Medium (DMEM, Life Technologies, Carlsbad, CA), 10% FBS, and 1% P/S at standard culture conditions (Yadav et al., 2012).

### 2.2 ECM production

MSCs were preconditioned with L-ascorbic acid-2-phosphate (Sigma-Aldrich, St. Louis, MO) and expanded for 7 d. For ECM production, preconditioned MSCs were seeded at 50,000 cells/cm^2^ in growth media or λ-carrageenan supplemented growth media [standard growth media with 75 μg/mL λ-carrageenan (MilliporeSigma, St. Louis, MO)]. After 9 d, MSC-secreted ECM was decellularized (dECM) and collected using one of two methods: (Gresham et al., 2021) a standard detergent removal decellularization method or (McKay et al., 2007) exposure to DNase alone. For the detergent removal protocol, cultures were incubated in a solution of 0.5% Triton X-100 in 20 mM ammonium hydroxide (NH_4_OH) for 5 min followed by treatment with 0.1 mg/mL DNase I (MilliporeSigma), both at 37 ° C, with three phosphate buffered saline (PBS) washes between each step. DNase decellularization was achieved by incubation in 0.1 mg/mL DNase I for 1 h at 37 ° C. After rinsing, ECMs were scraped with a cell scraper, collected in 0.02 N acetic acid, sonicated until homogeneous, and stored at −20 ° C (Decaris et al., 2012).

### 2.3 ECM characterization

We used protein mass spectrometry (MS) to analyze the composition of dECM (10 μg of 1 mg/mL solution in RIPA buffer). Protein was separated using a 10% Bis-Tris Novex mini-gel (Invitrogen, Waltham, MA) and Coomassie blue stain and analyzed by nano LC/MS/MS with a Waters M-Class HPLC system interfaced to a ThermoFisher Fusion Lumos (MSBioworks, Ann Arbor, MI) (Mitra et al., 2017; Harvestine et al., 2018). MS was performed on equal quantities of protein. MS data, obtained in spectral counts (SpC), was multiplied by a factor of 5.86 to account for the difference in total amount of protein between samples post-decellularization.

Reconstituted ECM samples were prepared for scanning electron microscopy (SEM) following a standard hexamethyldisilazane dehydration process (Mitra et al., 2017; Ramos-Rodriguez et al., 2021). Poly (lactic-co-glycolic acid) 50:50 (PLGA) thin filaments were made as supporting scaffolds to be coated with the dECM. PLGA scaffolds were fabricated by solvent casting with 15% w/w PLGA 50:50 solution in dichloromethane. Reconstituted dECM (10 µL) was pipetted on top of 4 mm PLGA discs and dried overnight. Samples were dehydrated with increasing concentrations of ethanol (35%, 50%, 75%, and 100%) for 15 min each. To complete the dehydration process, hexamethyldisilazane (HMDS) was used in 1:1 proportion with ethanol for 1 h followed by a 5 min incubation in pure HMDS. Samples were allowed to dry at room temperature (RT) inside a fume hood for 1 h. After dehydration, samples were mounted on aluminum pin stubs with double sided carbon tape to identify structural differences between ECM coatings. All samples were sputter coated with gold for 14 s at 40 mA in a Denton Vacuum Sputter Coater (Denton Vacuum, Moorestown, NJ) and imaged under a Quattro SEM (ThermoFisher, Waltham, MA). Voltage and spot size for all samples was 5 kV and 3.0 nm, respectively. Each sample was randomly imaged on at least 3 different spots at ×5000, ×8000, and ×20000 magnification.

Total protein was quantified via Pierce™ BCA Protein Assay Kit (ThermoFisher) per the manufacturer’s instructions. Briefly, ECM solutions were mixed with the provided working solution, deposited in a clear 96 well plate, incubated for 0.5 h at 37°C, and absorbance was quantified at 562 nm using a Synergy HTX multi modal plate reader (BioTek, Santa Clara, CA). DNA content was quantified via a Quant-iT™ PicoGreen assay (ThermoFisher) per the manufacturer’s instructions. Briefly, sonicated ECM solutions were spun at 5000x*g* to pellet out debris, and supernatant was diluted 1:4 in TE buffer. Samples were mixed 1:1 with PicoGreen working solution in a black 96 well plate, and fluorescence was quantified at 485/528 in the Synergy HTX plate reader. Total GAG content was quantified using a 40 µM 1,9-dimethyl-methylene blue zinc chloride double salt (DMMB) working solution [0.3% glycine (w/v)/0.16% sodium chloride (w/v)/0.01 M acetic acid] in water at a pH of 1.5. Samples were diluted with the working solution (1:10) in a clear 96 well plate, and absorbance at 525 nm was quantified against a known concentration of chondroitin sulfate (Zheng and Levenston, 2015).

### 2.4 BMP-2 adsorption to cell-secreted ECMs

Glass bottomed culture wells were coated with ECM at 3,000 μg/cm^2^ and air dried before BMP-2 adsorption. Recombinant human BMP-2 (PeproTech, Cranbury, NJ) was fluorescently labeled with DyLight 488 Microscale Antibody Labeling Kit (ThermoScientific). Coated plates were incubated with 100 ng of fluorescently labeled BMP-2 for 2 h in PBS and rinsed once with PBS before fluorescent readings (excitation: 493 nm, emission: 518 nm) on a Synergy HTX Multi-Mode Reader (BioTek, Winooski, VT).

BMP-2 dissociation constants were determined using a saturation isotherm as previously described (Bhat et al., 2011). Glass bottomed culture wells were coated with 3,000 μg/cm^2^ of MMC dECM, 3,000 μg/cm^2^ dECM, or 3,000 μg/cm^2^ rat tail collagen 1 as a control (Corning, Corning, NY). Plates were air dried and kept at 4°C until use. BMP-2 was diluted in PBS to 200 ng/μL, 100 ng/μL, 50 ng/μL, or 25 ng/μL. 50 µL of BMP-2 solution was deposited on protein coated plates for 2 h at RT. The BMP-2 solution was then collected, plates were washed with 100 µL of PBS, bringing the total collected supernatant volume to 150 µL. BMP-2 remaining in the supernatant was quantified using a BMP-2 ELISA (R&D Systems, Minneapolis, MN) per the manufacturer’s instructions and quantified in a Synergy HTX multi modal plate reader. The adsorbed BMP-2 was determined by subtracting the mass of BMP-2 in the supernatant from the initial amount deposited in the well. The bound BMP-2 mass was plotted against the concentration and a non-linear hyperbolic fit was utilized to determine the dissociation constant (K_D_) (Bhat et al., 2011).

### 2.5 Bioactivity of cell-secreted ECM

Glass bottomed culture wells were coated with ECM (3,000 μg/cm^2^) and air dried. Dried ECM was scraped in the presence of PBS, and endogenous BMP-2 was quantified using a BMP-2 ELISA (R&D Systems). For BRITER studies, 50 ng of BMP-2 was adsorbed onto each coating for 2 h at RT and washed once with PBS. BRITER osteoblasts were seeded onto coated wells at 60,000 cells/well in growth medium containing Gibco DMEM, 10% FBS, 1% P/S for 24 h. BRITER osteoblasts were then exposed to10x IVISbrite D-luciferin Potassium Salt (PerkinElmer, Waltham, MA) in DMEM for 10 min, and cell luminescence was captured by an IVIS^®^ Spectrum *In Vivo* Imaging System (PerkinElmer).

### 2.6 Ectopic model of bone formation by cell-secreted ECM

All animal procedures were performed under IACUC approval and in accordance with the Guidelines for Care and Use of Laboratory Animals of the National Institutes of Health and UC Davis. To explore the capacity of ECMs to promote bone formation in an ectopic site, we implanted ECMs into the muscle pouch of 13-week-old C57BL/6J male and female mice (Strain 000664, Jackson Laboratory, Bar Harbor, ME) (Asatrian et al., 2014). MMC dECM or dECM (100 µg) were deposited on a well-plate and allowed to air dry at RT. Once dry, some ECM samples were used to adsorb BMP-2. For BMP-2-complexed ECMs, 20 µg of BMP-2 was adsorbed for 2 h onto each dECM, rinsed twice before scraping, collected in PBS, and lyophilized overnight for implantation.

Anesthesia was induced by 3% isoflurane gas, and animals were maintained under 2% isoflurane anesthesia for the remainder of the procedure. Hair was removed from the right hind limb of the animal, and the limb was sterilized with ethanol and betadine wipes three times. Using sterile technique, a 5 mm linear incision was made on the posterior proximal hindleg, and blunt dissection was used to expose the fascia overlying the biceps femoris muscle belly. The fascia was sharply split, and scissors used to gently spread the muscle in line with its fibers to create a 5 mm × 2 mm pocket. Care was taken to minimize soft tissue disruption outside the muscle split and avoid dissection near the periosteum. Once the muscle pouch was created, the appropriate lyophilized dECM sample was placed in the space, and the overlying fascia closed with a single figure-of-eight vicryl stitch. Hydroxyapatite nanopowder (20 mg, Millipore Sigma) was suspended in 20 µL of sterile PBS, pelleted with centrifugation, and delivered into the muscle pouch as a positive mineral control occupying a similar volume as dECMs. The skin was closed with a staple. Mice received a weight-based injection of buprenorphine, and animals were placed back in their cages and observed until they exhibited full activity and recovery.

At 4 weeks post-op, mice were anesthetized via 3% isoflurane inhalation. Radiographs and planar x-rays were acquired using a Kubtec Parameter 3D Cabinet x-ray system (Mozart, Kubtec Medical Imaging, Stratford, CT), using the Digicom 11 3D tomosynthesis software (kV = 22, μA = 400). Serial 2D radiographs were generated as well as a composite image of all radiographs (slice thickness = 0.96 mm).

After imaging, mice were euthanized by CO_2_ inhalation, and the biceps femoris muscle was harvested. The skin overlying the operative hindleg, and one contralateral hindleg was removed. Tissue was carefully dissected, and the leg was mounted on light tension in anatomic alignment and fixed in 4% paraformaldehyde for 24 h at 4°C.

### 2.7 Assessment of mineral formation by microCT and histology

Whole biceps femoris explants were imaged at a voxel resolution of 10 µm on a high-resolution microCT scanner (mCT 35, Scanco Medical, Brütisellen, Switzerland). A cylindrical region 13.93 mm in diameter between the pelvis and the patella was analyzed due to variability in the placement of the dECM constructs in the biceps femoris. Quantification was performed with a threshold of 160–3,000 mg HA/cm^3^ to partition mineralized tissue from soft-tissue and liquid. Bone volume fraction was determined by dividing the number of voxels greater than the threshold (BV) by the total number of pixels in the volume (TV).

Fixed explants were rinsed with PBS and submerged in Calci-Clear (National Diagnostics, Atlanta, GA) for 7 d, with solution exchanges every 2 d. Tissues were washed, paraffin embedded, and sliced into 10 µm sections. Sections were rehydrated and exposed to heat mediated antigen retrieval using a 10 mM sodium citrate buffer (pH 6). Tissue sections were incubated in blocking buffer (10% goat serum and 10 mg/mL bovine serum albumin) for 1 h at RT. Slides were incubated with anti-osteocalcin antibody (Abcam, Cambridge, MA ab93876, 1:150) overnight at 4°C. Slides were then treated with a secondary goat anti-rabbit antibody conjugated to AlexaFluor 488 (Abcam, Cambridge, Ma, ab150083, 1:300) for 1 h at RT. Slides were counterstained with DAPI (ThermoFisher) and imaged using a ×20 objective on a confocal microscope (Stellaris, Leica Microsystems, Wetzlar, Germany).

### 2.8 Statistical analysis

All experiments were performed with at least three independent replicates, and data are presented as means ± standard deviation unless otherwise stated. Statistical analysis was performed by one-way ANOVA with multiple comparisons test or Student’s *t*-test as appropriate using Prism 9 software (GraphPad, San Diego, CA). Groups with different letters indicate significance (*p* < 0.05) while groups with the same letter are not significantly different.

## 3 Results

### 3.1 λ-carrageenan enhances glycosaminoglycan content and protein diversity in dECM

**FIGURE 1:**
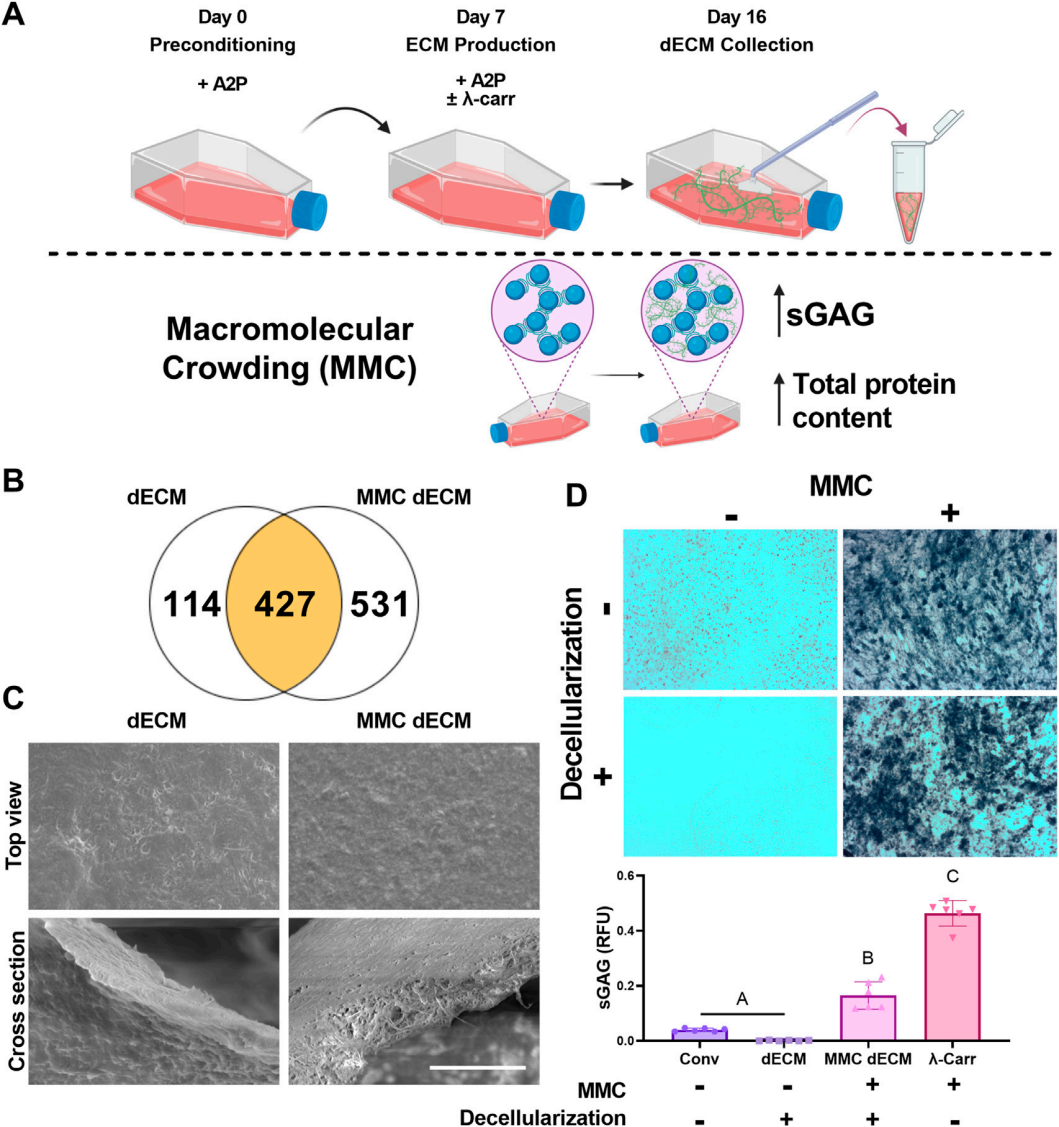
Macromolecular crowding increases sulfated glycosaminoglycan content within a cell-secreted extracellular matrix (ECM). **(A)** Schematic of ECM culture. **(B)** Mass spectrometry revealed the number of unique proteins within λ -carrageenan ECM (MMC dECM) compared to ECM produced without λ -carrageenan (dECM). **(C)** Topographical representation of ECM imaged via scanning electron microscopy. Scale bar is 5 μm (×20,000 magnification) **(D)** Alcian blue staining identified the presence of sulfated glycosaminoglycans (sGAG) in culture (top) and upon quantification of staining (bottom). MMC dECM was decellularized with DNase, while dECM was decellularized with Triton X-100 and DNase. Data are mean ± std., deviation (*n* = 6). Groups with different letters are significantly different determined by one-way ANOVA.

MSCs were seeded at confluency for ECM production in standard growth media or growth media supplemented with λ-carrageenan (Figure 1A). Upon analysis of dECM by mass spectrometry, dECM and MMC dECM had 427 proteins in common. We observed that culture with λ-carrageenan yielded an ECM (MMC dECM) with 531 unique proteins, while decellularized ECM generated in standard growth media (dECM) contained 114 unique proteins (Figure 1B). Compared to dECM, MMC dECM had more type I collagen (3.4x), type III collagen (5.7x), type V collagen (1.4x), fibronectin (4.7x), and vitronectin (2.9x) (Supplementary Table S1). However, we detected small quantities of biglycan and decorin in dECM that were undetected in MMC dECM. The relative contribution of collagen in dECM was similar to our prior study (Harvestine et al., 2018), yet we detected more collagen VI and less collagen V in both MMC dECM and dECM. The percentage of collagen to total protein in MMC dECM was 16.8%, while the overall collagen content in dECM was 37.9%. Differences are attributed to the increased production and retention of other macromolecules in MMC dECM compared to dECM.

Topographically, both dECM and MMC dECM exhibited fibrillar structures with mesh-like appearance and smooth surface coating (Figure 1C). Alcian blue staining revealed that λ-carrageenan increased sGAG content (Figure 1D). Upon quantification, supplementation with λ-carrageenan increased sGAG 11-fold (*p* < 0.0001), while the combination of λ-carrageenan and DNase decellularization resulted in a 134-fold sGAG increase (*p* < 0.0001) compared to dECM. Decellularization with DNase retained 36% of the initial sGAG in MMC dECM, while decellularization of ECM with both Triton X-100 and DNase resulted in only 3% retention of sGAG content. Overall, the addition of the MMC to the culture media enhanced protein and sGAG yield within dECM.

### 3.2 DNase decellularization retains sGAG content within dECM

**FIGURE 2:**
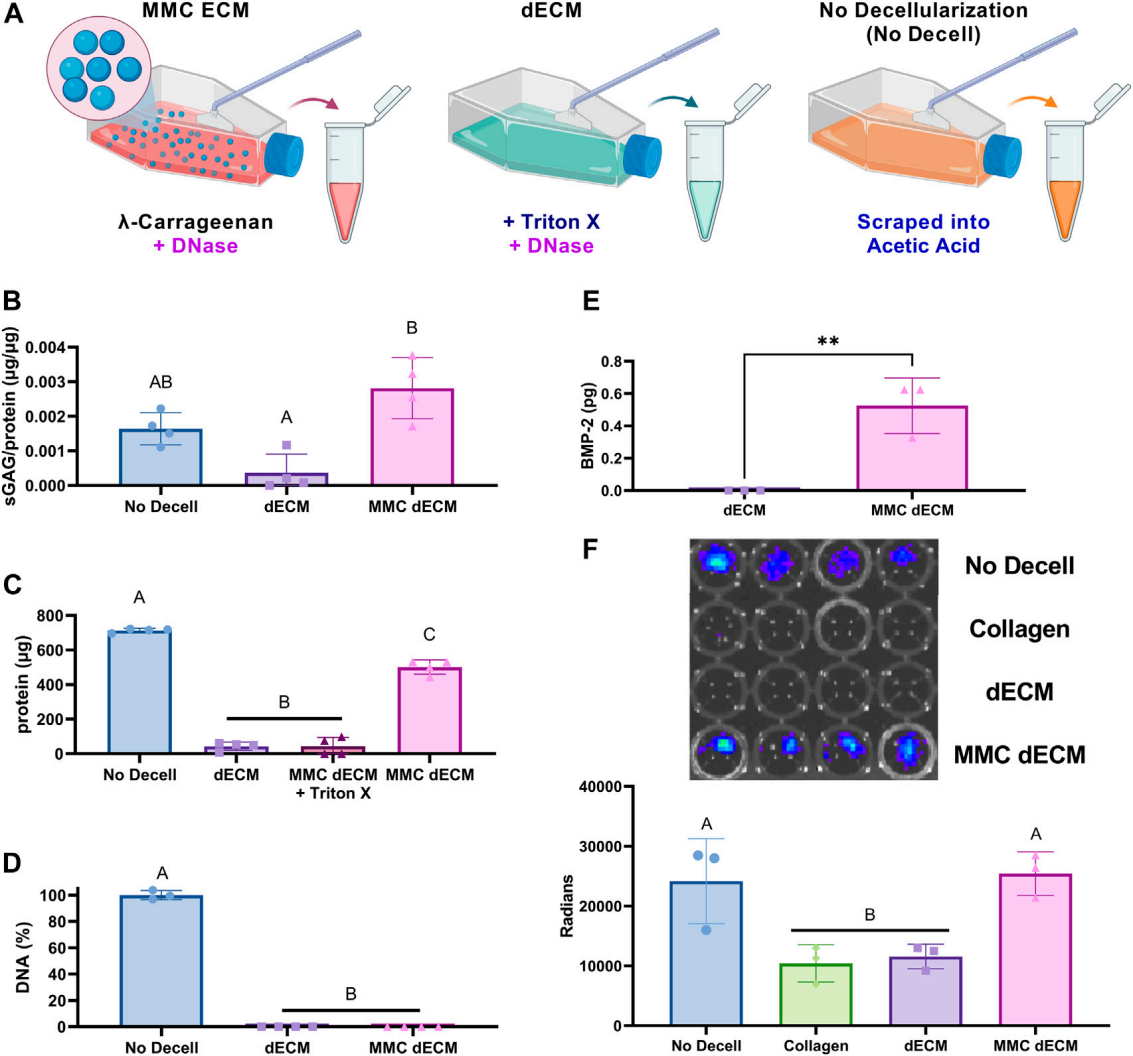
ECM yield and sulfated glycosaminoglycan content are influenced by addition of λ -carrageenan and decellularization method. **(A)** Schematic depicting culture and decellularization methods for ECM production. **(B)** Quantification of sGAG content within ECMs following dimethylmethylene blue (DMMB) assay. **(C)** Total protein content in ECMs. **(D)** Residual DNA content within ECMs. **(E)** Endogenous BMP-2 within the ECMs was detected directly via ELISA and indirectly with **(F)** luminescence of BRITER cells (top) and quantification (bottom). Data are mean ± SD (*n* = 3–4). Groups with different letters are significantly different determined by one-way ANOVA or Student’s *t*-test as appropriate, **p < 0.01.

To increase sGAG content and retention in dECM, we investigated the effect of both MMC and DNase decellularization compared to our previously reported detergent-based decellularization method (Figure 2A). As a positive control for endogenous BMP-2 detection, we used cultures with no MMC and no decellularization reagent that were subsequently scraped, collected in acetic acid, lyophilized, sonicated, and denoted as “No Decell.” MMC and DNase decellularization yielded dECM with significantly greater sGAG and protein synthesis as evidenced by the 7.7-fold sGAG increase (*p* = 0.0013) (Figure 2B) and 11.7-fold increase in total protein content (*p* < 0.0001) (Figure 2C) compared to dECM. For MMC ECMs prepared with λ-carrageenan, we detected an 11.5-fold decrease (*p* < 0.0001) in total protein when decellularizing with Triton-X-100 compared to those decellularized with DNase alone. These data reveal the deleterious effects of detergent-based decellularization on protein yield.

DNA removal during tissue decellularization is essential for avoiding the heightened immune reaction to foreign components, and the efficiency of DNA removal is a key indicator of successful decellularization. We quantified the amount of residual DNA in the ECM and found no significant differences between our conventional detergent-based removal decellularization with Triton X-100, which removes greater than 98% of DNA, compared to DNase alone (Figure 2D). Additionally, MMC dECM with DNase decellularization retained endogenous BMP-2 as detected via ELISA (Figure 2E) and luminescence of BMP-2 responsive (BRITER) cells (Figure 2F). The BRITER calvarial osteoblast is an immortalized population based on a tamoxifen-induced double knockout of BMP-2 and BMP-4 (Yadav et al., 2012). These osteoblasts, when exposed to luciferin, exhibit robust luminescence in the presence of exogenous BMP through the activation of BMP responsive units driven primarily by SMAD phosphorylation. Luminescence of BRITER cells on MMC dECM was 2-fold greater than cells on dECM (*p* = 0.011), while there were no significant differences between MMC dECM and the No Decell control. Luminescence indicates that more BMP was retained in the extracellular matrix of the MMC dECM and the No Decell control groups. These data demonstrate that supplementation of culture media with MMC and decellularization with DNase alone effectively increases and retains more sGAGs than detergent-based decellularization. Moreover, the use of DNase as a decellularization agent better preserves bioactive endogenous growth factors within the ECM.

### 3.3 sGAGs are functional in MMC ECM and improve growth factor adsorption

**FIGURE 3:**
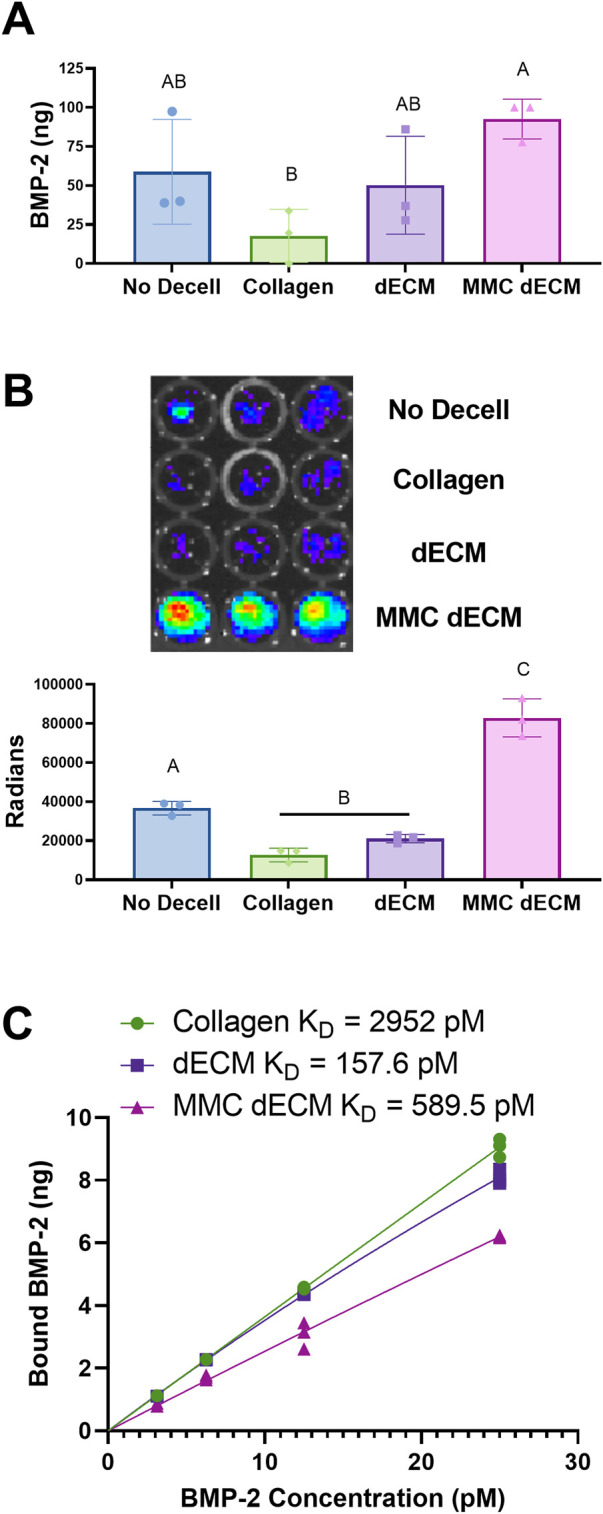
BMP-2 adsorption is increased on ECMs formed with macromolecular crowding agent. **(A)** Quantification of adsorption of fluorescently labeled BMP-2 on each ECM. **(B)** Luminescence of BRITER cells in response to 50 ng of adsorbed BMP-2. **(C)** BMP-2 saturation binding curves of rat tail collagen 1, dECM, and MMC dECM. Data are mean ± SD (*n* = 3). Groups with different letters are significantly different determined by one-way ANOVA.

We evaluated BMP-2 adsorption directly using fluorescently labeled BMP-2 and indirectly by measuring BRITER cell luminescence. After incubation with 100 ng of fluorescently labeled BMP-2 for 2 h, we observed a 2.8-fold increase in adsorption on dECM (*p* = 0.44) and a 5.2-fold increase in adsorption on MMC dECM (*p* = 0.028) compared to collagen controls (Figure 3A). We also explored whether we would observe a bioactive response of cells to a lower dose of BMP-2 on decellularized ECMs. In the presence of 50 ng BMP-2, BRITER cells were 6.5-fold (*p* < 0.0001) more luminescent on MMC dECM compared to cells on collagen, while cells on dECM exhibited a 1.6-fold increase in luminescence (*p* = 0.32). When comparing BRITER cell luminescence on ECM coatings, cells on MMC dECM exhibited a 3.9-fold increase in luminescence (*p* < 0.0001) compared to cells on dECM (Figure 3B). These data confirm that exogenous BMP-2 is more efficiently adsorbed to MMC dECM compared to dECM or collagen controls, likely due to the increased presence of sGAGs achieved by culture with a MMC agent and decellularization method.

Fitting saturation isotherms with hyperbolic fits, we identified dissociation constants (K_D_) values of 2952 pM for collagen 1, 157.6 pM for dECM, and 589.5 pM for MMC dECM (Figure 3C). The higher K_D_ of MMC dECM compared to dECM indicates less affinity but is likely due to the retention of endogenous proteins occupying the sulfated sites on the ECM. The improved response of BRITER cells and retention of BMP-2 *in vitro* justified further investigation of this mechanism in an *in vivo* model.

### 3.4 MMC dECM improves mineralized tissue formation in a murine muscle pouch model

**FIGURE 4:**
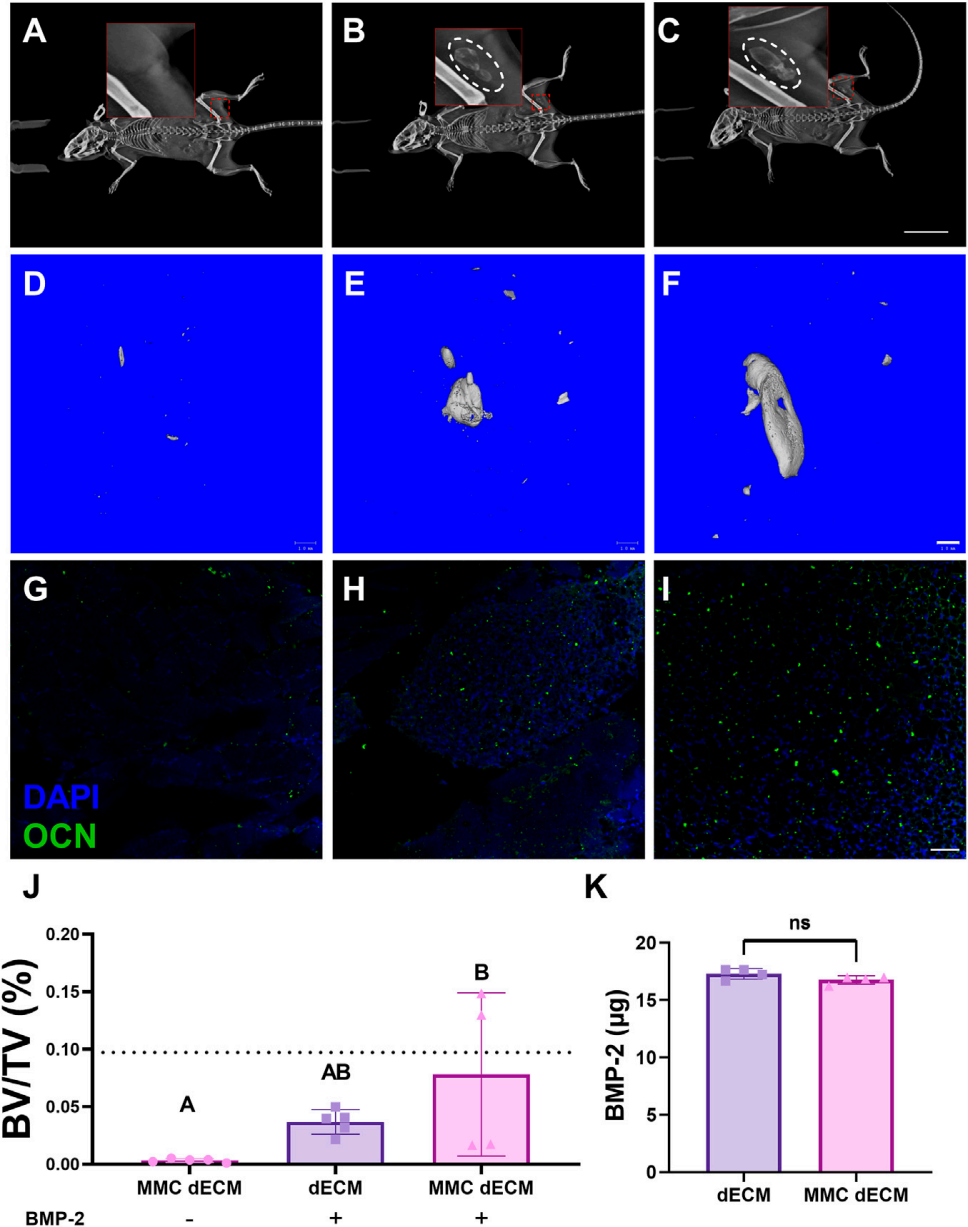
Mineralized tissue is increased in the murine muscle pouch when implanted with MMC dECM and adsorbed BMP-2. **(A–C)** Radiographs were taken at day 28 in live animals. Hashed white circles denote areas with mineralized tissue in **(A)** MMC dECM, **(B)** dECM + BMP-2, and **(C)** MMC dECM + BMP-2. Scale bar is 20 mm. **(D–F)** MicroCT reconstruction reveals mineralized tissue generated in **(D)** MMC dECM, **(E)** dECM + BMP-2, and **(F)** MMC dECM + BMP-2. Scale bar is 1 mm. **(G–I)** Tissue sections stained for osteocalcin (OCN, green) and DAPI (blue) in biceps femoris containing **(G)** MMC dECM, **(H)** dECM + BMP-2, and **(I)** MMC dECM + BMP-2. Scale bar is 50 µm. **(J)** Quantification of bone volume fraction in the interrogated volume. Dotted lines denote the mean value of HA groups. **(K)** Quantification of BMP-2 adsorbed to ECMs for *in vivo* study. Data are mean ± SD (*n* = 4–5). Groups with different letters denote statistical significance as determined by one-way ANOVA or Student’s *t*-test as appropriate.

To evaluate the capacity of MMC dECM to promote mineralized tissue formation, we implanted ECMs with or without BMP-2 in the muscle pouch of C57BL/6J mice and assessed mineralized tissue formation after 4 weeks. Radiographic assessment of live specimens revealed dense tissue areas in the operated limb in all groups except for the MMC dECM lacking BMP-2 (Figures 4A–C). Assessment of fixed biceps femoris tissue via microCT corroborated this increase in mineralized tissue (Figures 4D–F). Bone volume fraction was highest for MMC dECM with adsorbed BMP-2, with 100% more mineralized tissue in the MMC dECM compared to dECM with BMP-2 (Figure 4J). Furthermore, we observed the greatest osteocalcin expression in BMP-2 presenting MMC dECM (Figures 4G–I). The comparable initial adsorption of BMP-2 between the ECM substrates (Figure 4K) confirms that increased ossification is not solely due to increased BMP-2 retention. These data indicate that BMP-2 adsorbed to MMC dECM increased osseous tissue formation *in vivo*.

## 4 Discussion

Growth factor delivery is a promising alternative to autologous bone grafts or other implants for bone regeneration, yet challenges persist with timing and dosages necessary to achieve the desired effect (Gresham et al., 2021). For example, current treatments release milligram quantities of BMP-2 from collagen sponges, which can cause adverse side effects due to high dosages and uncontrolled diffusion of BMP-2 (McKay et al., 2007). Collagen provides low binding specificity and retention, resulting in a burst release of any intended growth factor payload (Yamamoto et al., 2003). There is a critical need to develop biomaterials that increase the efficient delivery of low growth factor dosages and achieve the desired therapeutic outcome (Visser et al., 2009; Ren et al., 2019; Gresham et al., 2021). We previously engineered an instructive, MSC-secreted ECM that could be efficiently decellularized and used as a culture platform or cell delivery vehicle (Decaris and Leach, 2011; Decaris et al., 2012; Bhat et al., 2013). However, we have not assessed the ability of this decellularized ECM to retain growth factors nor did we previously detect the retention of endogenous growth factors. Within the native ECM, sGAGs bind heparin-binding growth factors such as BMP-2 through N-termini interactions for local presentation to associated cells. The conformational plasticity of BMP-2 and its interaction with sGAGs play a key role in the stabilization and prolonged half-life of BMP-2, which enhances its effect on osteogenic differentiation (Zhao et al., 2006; Hintze et al., 2014). Techniques that increase the generation and retention of sGAGs offer the ability to deliver growth factors more efficiently for tissue generation.

Numerous approaches to increase the sGAG-content in engineered or native ECMs have been pursued including physical or chemical crosslinking via UV light, temperature, pH, carbodiimide chemistry (Neves et al., 2020), addition of serine-rich peptides for increased sGAG retention (Miles et al., 2016), or modification of ECMs with proteoglycan-mimicking sulfate groups (Gionet-Gonzales et al., 2021). Macromolecular crowding has been employed to better recapitulate the physiological environment and increase protein synthesis (Martínez-Vidal et al., 2022). The addition of an MMC agent into cell culture medium enhances ECM deposition by creating volumes of reduced space, increased chemical potential, and resultant increases in effective concentration (Graham et al., 2019; Badowski et al., 2020; Tsiapalis and Zeugolis, 2021). While previous studies assessed the effect of MMC on protein composition, cellular processes, and differentiation (Cigognini et al., 2016), we sought to employ MMC to increase production and retention of sGAGs in the ECM for the delivery of growth factors.

To exploit the potential of a biologically relevant matrix as a growth factor delivery vehicle, we combined λ-carrageenan as an MMC with DNase decellularization to generate ECMs with increased sGAG content. These studies were designed to exclude any approach that does not preserve sGAG content while simultaneously eliminating DNA content, which is essential for any effective decellularization technique. λ-carrageenan is a widely available red algae-derived polysaccharide used in higher quantities as a thickener and stabilizer in commercial food products. To our knowledge, the use of λ-carrageenan as an MMC agent on cell-secreted ECM as a biomaterial for growth factor delivery has not been reported. Compared to previous reports of dECM composition (Harvestine et al., 2018), MMC dECM contained a more diverse collection of macromolecules. The simple addition of an MMC agent during cell culture yielded an ECM enriched in sGAGs capable of sequestering and retaining heparin-binding growth factors. Furthermore, decellularization with only DNase mitigated sGAG loss that is common with detergent based methods (Crapo et al., 2011). These data highlight the synergistic effect of supplementation with MMC and decellularization methods on protein and sGAG composition and DNA removal in decellularized matrices. While the increased presence of sGAG is evident, it is unclear whether MMC contributes to sGAG retention. The mechanism requires further investigation to fully realize the potential of this approach.

Compared to single-component biomaterials, cell-secreted ECMs contain a complex network of macromolecules that retain their native instructive potential and may overcome the variability of tissue-derived matrices. Our findings demonstrate that MMC dECM contains endogenous BMP-2, consistent with reports of other decellularized tissues retaining growth factors (Fercana et al., 2017). However, it is not clear if this BMP-2 presentation is due to the MMC culture environment or the different decellularization process. It is likely that other endogenous growth factors may be retained in this matrix, but our quantification method was not sensitive enough to detect them in measurable quantities. While the retention of endogenous BMP-2 is exciting, we did not observe significant increases in the adsorption of exogenous BMP-2 to MMC dECM compared to dECM or collagen. The sGAGs may be occupied with other heparin-binding growth factors, obscuring available sites for exogenous growth factor binding. The availability of growth factor binding sites in cell-secreted ECMs warrants further investigation. Moreover, the adhesion of MMC ECM to culture surfaces was tenuous due to the increased hydrophobicity associated with a sGAG-rich construct. Improvements or modifications for dECM adherence to surfaces for use as a coating may be explored. MMC ECM’s ability to sequester growth factors opens new avenues for the delivery of other heparin-binding growth factors. For example, transforming growth factor beta-1 (TGF-β1) is a heparin-binding macromolecule that plays a key role in chondrogenesis and cartilage formation (McCaffrey et al., 1992). The successful delivery of TGF-β1 for cartilage regeneration faces challenges due to toxicity when delivered systemically (Prud'homme GérJ, 2007). Thus, MMC-modification of a cell-secreted ECM provides an exciting opportunity for controlled delivery of TGF-β1 for cartilage regeneration. Additionally, the complexation of heparin-binding moieties to non-heparin-binding growth factors such as Insulin-Like Growth Factor (IGF) facilitates the use of sGAG for local delivery (Florine et al., 2015).

Naturally derived biomaterials have been widely explored to capture the characteristics of native tissue and promote tissue regeneration. Among others, insoluble collagenous bone matrix (ICBM) and demineralized bone matrix (DBM) have been successfully used to promote bone formation (Ripamonti et al., 1993). These materials require the harvest and demineralization of allogeneic bone. This approach is limited by tissue availability and potential immunogenicity, further motivating the exploration of cell-secreted matrices for bone regeneration. In this study, we observed increased tissue mineralization in an ectopic site and increases in non-collagenous proteins associated with osseous tissue formation. Importantly, the MMC dECM and dECM groups exhibited similar initial adsorption of BMP-2, yet implantation of MMC dECM resulted in greater bone volume. This finding suggests that the generation of osseous tissue may be driven by slower release of BMP-2 *in vivo* or perhaps additional endogenous factors that are beneficial to ossification. Although the mineralized tissue was relatively immature at 4-weeks post implantation, we anticipate a more developed tissue structure would result over longer durations. Further, osseous tissue formation in this ectopic site is dependent upon cells with low osteogenic potential that are incapable of generating a hematopoietic niche such as fibro-adipogenic progenitor cells (Lemos et al., 2015). Upon implantation in an orthotopic site, we would expect more mineralized tissue due to the presence of endogenous osteoinductive cues and bone-forming progenitor cells.

## 5 Conclusion

These studies demonstrate that the addition of λ-carrageenan as a macromolecular crowder in conjunction with detergent-free decellularization increases the incorporation and/or retention of sulfated glycosaminoglycans within a decellularized matrix. Sulfated glycosaminoglycans improve retention of endogenous BMP-2 or adsorption of exogenous BMP-2 for greater mineralized tissue formation. These data establish the importance of macromolecular crowding and decellularization method on the generation of bioactive MSC-secreted ECMs. These strategies represent an exciting platform for the generation of specific ECM constructs for tissue engineering and regeneration.

## Data Availability

The original contributions presented in the study are included in the article/Supplementary Material, further inquiries can be directed to the corresponding author.

## Abbreviations

BMP-2, Bone morphogenetic protein 2; BRITER, BMP responsive immortalized reporter cell line; DBM, Demineralized bone matrix; DMMB, Dimethylmethylene Blue; dECM, Decellularized extracellular matrix; ECM, Extracellular matrix; IGF, Insulin-like growth factor; ICBM, Insoluble collagenous bone matrix; MMC, Macromolecular crowder; OCN, Osteocalcin; sGAG, Sulfated glycosaminoglycan; TGF-β1, Transforming growth factor beta-1.
